# Cross-Market Risk Spillovers in China: A Multidimensional and Nonlinear Transmission Perspective

**DOI:** 10.3390/e28101110

**Published:** 2026-10-11

**Authors:** Yijiang Zou, Qinghua Chen, Jihui Han, Longfeng Zhao

**Affiliations:** 1 School of Economics, Anyang Normal University, Anyang 455000, China; zouyj@mails.ccnu.edu.cn; 2 School of Systems Science, Beijing Normal University, Beijing 100875, China; 3 School of Computer Science and Technology, Zhengzhou University of Light Industry, Zhengzhou 455001, China; hanjihui@zzuli.edu.cn; 4 School of Management, Northwestern Polytechnical University, Xi’an 710072, China; zlf@nwpu.edu.cn

**Keywords:** systemic risk, connectedness network, non-linear transmission, effective transfer entropy, quantile VAR, key market segments

## Abstract

This paper investigates the non-linear characteristics and heterogeneous evolution of cross-market risk transmission in China by constructing a multi-layered analysis framework that integrates linear spillovers, non-linear transmission, and tail risk diffusion. By employing the Diebold–Yilmaz spillover index, effective transfer entropy (ETE), and quantile vector autoregression (QVAR), we systematically measure the dynamic connectedness structures and transmission pathways across key financial markets and strategic real-economy sectors. Our empirical results yield several critical insights. First, the Chinese cross-market risk network exhibits significant state-dependence and asymmetric features, where negative shocks generate stronger spillover intensities than positive ones, and systemic connectedness intensifies substantially under extreme quantile conditions, demonstrating pronounced non-linear amplification effects. Second, the high-tech sector acts as a persistent net source of non-linear information flow, which is consistent with the influence of industrial technology cycles on financial markets rather than constituting direct causal evidence. Third, the real estate market displays high network centrality, operating as a predominant net risk receiver and the ultimate heavy-bearing terminal of systemic stress. Finally, time-varying network analysis reveals that major external shocks markedly elevate network density and overall coupling, triggering synchronized risk resonance. These findings unveil the structural mechanics of risk diffusion under emerging market conditions, offering novel empirical evidence and structural insights for macroprudential regulation and cross-market risk governance.

## 1. Introduction

With the deepening process of global economic integration, risk transmission across financial markets has evolved from localized “contagion” into a globalized “network effect”. Over the past decade, successive macroeconomic shocks, ranging from geopolitical turbulence to global health crises, have not only intensified the volatility of single assets but have also profoundly reshaped the co-movement mechanisms among core markets, including stock, bond, foreign exchange, energy, real estate, and commodity markets. Against this backdrop of high interconnectedness, accurately identifying risk spillover pathways and their dynamic evolutionary characteristics under varying market stress conditions is of paramount importance. This understanding is critical both for investors optimizing asset allocation and for regulatory authorities safeguarding against systemic risks [1].

Particularly in the context of China’s economic structural transformation and the two-way opening up of its financial markets, cross-market risk linkages exhibit increasingly complex structural characteristics. On the one hand, the Chinese real estate market is undergoing a profound process of deleveraging and asset revaluation. Its dual attributes as collateral and financial intermediary assets make it highly susceptible to becoming a terminal bearing sector for the non-linear convergence of systemic risk and vulnerability exposure [2]. On the other hand, as a new growth engine of China’s economy, the high-tech sector experiences price fluctuations driven not only by industrial technology cycles but also by the dual impacts of geopolitical factors and external technological constraints. This deeply intertwined “financial-real economy”structural relationship causes the risk spillover pathways in China’s financial system to display asymmetry and strong coupling characteristics distinct from developed markets, thereby intensifying network-based risk diffusion mechanisms.

However, traditional linear correlation analyses or mean-based econometric models can no longer adequately capture the complex dynamic structure of modern financial systems. As Baur [3] pointed out, market dependence structures exhibit significant asymmetry at different quantile levels, implying that analytical frameworks based on conditional means systematically underestimate the intensity of risk contagion under extreme scenarios. Although existing studies have begun introducing non-linear methods to characterize risk spillover mechanisms, their focus remains constrained. For instance, researchers frequently utilize information theory tools like transfer entropy to analyze cross-market information flows and regime switching. While these analyses explore diverse perspectives—such as the joint time-frequency domain [4,5], extreme volatility or specific political shocks [6], and multi-asset dynamic connectedness [7,8]—the current literature remains primarily focused on bivariate linkages or structural mappings of localized sub-markets. It fails to systematically characterize, within a unified framework, the overall evolutionary mechanism of multi-asset, multi-layered financial networks that incorporate technological expectations and real-economy vulnerabilities.

Methodologically, although the Quantile Vector Autoregression (QVAR) model proposed by Ando [9] provides an essential tool for characterizing tail dependence structures, its comprehensive application within a cross-asset, full-network framework remains relatively limited. Specifically, when the energy market encounters supply shocks [10] or the real estate market undergoes structural adjustments [11], how these localized shocks propagate across asset classes and evolve into systemic risks—including their transmission direction, intensity, and heterogeneity across different quantile states [12,13] still lacks a unified empirical framework for systematic explanation.

Motivated by these limitations, this paper constructs an integrated cross-market risk analysis framework to characterize the risk connectedness structure and dynamic evolutionary mechanisms among six core markets in China. Compared with existing literature, the marginal contributions of this study are reflected in the following three aspects: First, by simultaneously incorporating stock, bond, foreign exchange, real estate, energy, and high-tech markets, we establish a more comprehensive cross-asset risk network analysis system, thereby transcending traditional single- or dual-market analytical frameworks. Second, by combining quantile dependence structures with non-linear information transmission methods, we not only measure the intensity of risk spillovers but also unveil the directionality and asymmetry of information flows. Finally, through a systematic comparison of different market regimes (normal versus extreme scenarios), we precisely identify the leading driving sources and terminal receivers of cross-market risks, offering robust empirical evidence and structural policy insights for dynamic asset allocation optimization and macroprudential regulatory design.

## 2. Literature Review

With the deepening of financial liberalization and market integration, risk transmission among different markets has increasingly exhibited pronounced cross-market, cross-industry, and cross-asset characteristics. Particularly under the impact of extreme events, markets such as stocks, real estate, energy, and foreign exchange often display heightened co-movement, leading to a rapid contagion of systemic risk. A systematic review of relevant literature helps clarify the research positioning and marginal innovations of this study.

### 2.1. Evolution of Cross-Market Risk Spillover Research

The theoretical foundation of cross-market risk spillover research traces back to the financial contagion theory. Forbes and Rigobon [14] defined financial contagion as a significant increase in cross-market linkages during crisis periods, distinguishing it from interdependence under normal market regimes. From an asset pricing perspective, Kodres and Pritsker [15] proposed a “rational contagion” model, arguing that heterogeneous exposure to common macroeconomic risk factors serves as the primary channel for risk propagation. Conversely, Kyle and Xiong [16] focused on investor behavior, demonstrating how wealth constraints and amplification via forced liquidations induce cross-market co-movements. In the domain of systemic risk measurement, the Marginal Expected Shortfall (MES) proposed by Acharya et al. [17] and the Conditional Value-at-Risk (CoVaR) constructed by Adrian and Brunnermeier [18] have become benchmark methodologies for quantifying institutional contributions to systemic risk.

The spillover index framework developed by Diebold and Yilmaz [19,20,21], which integrates Vector Autoregressions (VAR) with directed network analysis, has emerged as a representative methodology in cross-market risk spillover literature. A vast body of empirical research has utilized this framework to investigate risk transmission across stock, commodity, energy, foreign exchange, and bond markets. For instance, Creti et al. [22] found that the connectedness between stock and commodity markets intensified significantly following the global financial crisis. Arouri et al. [23] highlighted the net risk exporting characteristics of oil markets toward equity sectors, while Greenwood-Nimmo et al. [24] mapped the structural network of risks within foreign exchange markets. Regarding the Chinese financial system, Zhou et al. [25] examined the spillover effects between domestic and international equity markets; Li and Giles [26] analyzed the time-varying correlations between stock and real estate sectors; and Xiao et al. [27] focused on the asymmetric impacts exerted by the energy sector on specific equity blocks. More recently, Liu et al. [1] extended this paradigm to G20 economies, constructing a tail event-driven network to highlight the predominant role of stock markets in contributing to aggregate systemic risk while pointing out the risk diversification potential of currencies like CNY.

The nexus between the real estate market and the broader financial system represents another critical research stream. Case et al. [28] demonstrated that housing market fluctuations propagate across markets through collateral and credit channels. Liow et al. [11] further revealed that the risk connectedness between global REITs and stock markets escalates drastically during crisis periods. Along this line, Caporin et al. [29] confirmed shift-contagion characteristics between real estate and financial markets using a Bayesian quantile-on-quantile approach, whereas Gong et al. [2] provided cross-country evidence that global economic policy uncertainty spillovers significantly reduce housing market returns, particularly during developed market downturns. Although these studies provide substantial insights into cross-market risk linkages, most existing literature remains constrained to traditional financial assets, leaving the rapidly emerging high-tech market under-investigated.

### 2.2. Non-Linear Transmission and Tail Risk Analysis

Concurrently with the deepening understanding of market microstructures, researchers have turned their attention to the non-linear features of risk transmission. The information-theoretic Transfer Entropy (TE) approach proposed by Schreiber [30] is capable of capturing non-linear causal relations without prior functional assumptions, and the Effective Transfer Entropy (ETE) further developed by Marschinski and Kantz [31] effectively eliminates small-sample biases. Dimpfl and Peter [32] applied transfer entropy to analyze non-linear information transmission between stock index futures and spot markets. In global real estate networks, Ji et al. [33] introduced an entropy-based network approach to unveil pairwise net transfer entropy and directional information flows among international housing segments. In evaluating these information flows, He and Shang [4] provided a systematic comparison of various transfer entropy measures in financial time series. More recently, regarding external macro shocks, Xiao et al. [34] utilized ETE to precisely map the non-linear and asymmetric spillover pathways of oil price uncertainty onto Chinese sector-level stock returns. Transitioning to policy-driven flows, Yao et al. [35] constructed a quantile transfer entropy model to unveil the heterogeneous information transmission between climate policy uncertainty and the Chinese new energy market under extreme tail states.

In terms of tail risk analysis, quantile regression methods have been widely adopted. The seminal work of Koenker and Bassett [36] laid the methodological foundation, which was subsequently extended to multivariate systems through the Quantile Vector Autoregression (QVAR) model introduced by Ando et al. [9]. Baur [3] proposed an alternative framework utilizing quantile regression to decompose dependence structures, providing a detailed mapping of dependencies across asymmetric and non-linear spectrums. Cross-market empirical studies by BenSaïda et al. [37] confirmed the ubiquity of the “bad news contagion” effect. Similarly, Baruník et al. [38] constructed separate spillover networks for good and bad news, revealing that negative shocks propagate with higher asymmetric efficiency; Wen et al. [39] identified consistent empirical regularities within the Chinese commodity and equity markets. Crucially, within the contemporary Chinese context, Liu et al. [12] applied the QVAR method to reveal that extreme conditions markedly amplify the asymmetric spillovers of policy uncertainties onto stock, commodity, and foreign exchange markets, a behavior similarly verified by Nasir et al. [13] who documented heightened return connectedness under bullish and bearish regimes compared to normal market conditions.

Recent work has further documented the nonlinear and heavy-tailed nature of Chinese equity returns, including systematic departures from normality, distinct topologies between anti-correlation and positive-correlation networks, and greater risk-sensitivity of negative-tail local Gaussian correlation networks relative to conventional Pearson networks [40,41,42]. These studies share our recognition that mean-based measures understate asymmetric and tail-specific co-movement. However, they operate within single-market stock universes, whereas this paper spans six heterogeneous asset classes and integrates linear DY spillovers, nonlinear ETE, and quantile-based tail connectedness to examine cross-market systemic risk transmission.

### 2.3. Risk Propagation Through the Lens of Network Topology

Complex network theory offers a fresh analytical paradigm for cross-market risk transmission. Billio et al. [43] early applied network methods to financial institutional connectedness, observing that network density surges markedly during crises. This phenomenon is further reinforced by Mensi et al. [44], who illustrated that G7 REIT network connectedness experienced abrupt spikes during the first wave of the COVID-19 outbreak before declining with vaccination success. Demirer et al. [45] constructed a global equity volatility connectedness network, unveiling a distinct “core-periphery” topology. Network centrality metrics, such as PageRank [46] and eigenvector centrality [47], have become essential for identifying systemically important nodes, while the network disparity filter proposed by Serrano et al. [48] enables the extraction of the multi-scale backbone from high-dimensional connectedness matrices.

Focusing on the Chinese market, Wang et al. [49] mapped sector-level risk spillover networks and identified the financial and industrial sectors as highly central. Wu et al. [50] employed graph theory alongside advanced time-series techniques to isolate systemically important sectors and track their time-varying spillover patterns. By integrating the CoVaR approach with PageRank, Zheng et al. [10] mapped the tail risk network of China’s energy system, confirming that exogenous shocks dynamically alter transmission topologies over time. Dynamic network analysis further suggests that under major event shocks, network connectedness exhibits “impulse-like” spikes, leading to a comprehensive exposure of systemic risk [51].

The intersection of high-tech industries and risk networks constitutes a burgeoning frontier. Guo et al. [52] documented that fintech and cryptocurrencies exert increasingly significant shocks on traditional financial markets. Chen and Pantelous [53] revealed that during the US-China trade friction, the high-tech sector’s role as a risk exporter became heavily pronounced. Shahzad et al. [54] analyzed the directional spillovers and connectedness among financial globalization, trade globalization, high-tech sectors, and China’s environmental footprint. Nevertheless, the systematic role of the high-tech market within medium-to-long-term, multi-market risk networks remains to be thoroughly investigated.

## 3. Methodological Framework

Based on the multi-layered analytical framework of “linear spillovers—non-linear transmission—tail risk diffusion” proposed in this study, this section systematically delineates the three core econometric methodologies utilized: the Diebold–Yilmaz spillover index, Effective Transfer Entropy (ETE), and the Quantile Vector Autoregressive (QVAR) model [9,20,21,30]. These three methodologies progress in a hierarchical manner to unveil the cross-market risk transmission mechanisms in China from three distinct dimensions: linear conditional mean spillovers, non-linear information transmission, and extreme tail risk co-movements.

### 3.1. Diebold–Yilmaz Spillover Index Framework

The spillover index framework proposed by Diebold and Yilmaz [20,21] is built upon the Generalized Forecast Error Variance Decomposition (GFEVD) within a Vector Autoregressive (VAR) model [19,55]. It quantifies the intensity of risk spillovers by measuring the extent to which forecast error variances of each market are contributed by volatilities originating from other markets. Because this method bypasses the need for orthogonalization, it effectively overcomes the traditional Cholesky decomposition’s sensitivity to variable ordering, making it highly suitable for multi-market linkage analysis.

#### 3.1.1. VAR Model Specification

Consider an *N*-variable Vector Autoregressive model of order *p*, denoted as VAR(*p*):

(1) $$X_{t}=\sum_{i=1}^{p}\Phi_{i}X_{t-i}+\varepsilon_{t},\;\varepsilon_{t}∼\left(0,\Sigma \right)$$

where $X_{t}={\left(x_{1,t},x_{2,t},\ldots ,x_{N,t}\right)}^{\prime }$ represents an N×1 vector of market returns, $\Phi_{i}$ denotes the N×N coefficient matrices, $\varepsilon_{t}$ is a vector of independently and identically distributed white noise shocks, and Σ is the corresponding covariance matrix.

Provided that the system satisfies the stationarity conditions, the VAR(*p*) process in Equation (1) can be inverted into an infinite-order Vector Moving Average, VMA(*∞*), representation:

(2) $$X_{t}=\sum_{j=0}^{\infty }A_{j}\varepsilon_{t-j}$$

where the N×N coefficient matrices $A_{j}$ obey the recursive relation $A_{j}=\Phi_{1}A_{j-1}+\Phi_{2}A_{j-2}+\ldots +\Phi_{p}A_{j-p}$, with $A_{0}=I_{N}$ (the N×N identity matrix) and $A_{j}=0$ for all j<0.

#### 3.1.2. Generalized Forecast Error Variance Decomposition

Utilizing the GFEVD framework, the generalized contribution of variable *j* to the *H*-step-ahead forecast error variance of variable *i* is defined as follows:

(3) $$\theta_{ij}\left(H\right)=\frac{\sigma_{jj}^{-1}\sum_{h=0}^{H-1}{\left(e_{i}^{\prime }A_{h}\Sigma e_{j}\right)}^{2}}{\sum_{h=0}^{H-1}\left(e_{i}^{\prime }A_{h}\Sigma A_{h}^{\prime }e_{i}\right)}$$

where $\sigma_{jj}$ is the *j*-th diagonal element of the covariance matrix Σ (representing the variance of the error term for the *j*-th equation), and $e_{i}$ is a selection vector with 1 as its *i*-th element and 0 otherwise. To ensure that the sum of elements in each row of the variance decomposition matrix equals unity, the entries are normalized as follows:

(4) $${\tilde{\theta }}_{ij}\left(H\right)=\frac{\theta_{ij}\left(H\right)}{\sum_{j=1}^{N}\theta_{ij}\left(H\right)}$$

By construction, these normalized entries satisfy $\sum_{j=1}^{N}{\tilde{\theta }}_{ij}\left(H\right)=1$ and $\sum_{i,j=1}^{N}{\tilde{\theta }}_{ij}\left(H\right)=N$.

#### 3.1.3. Construction of Spillover Indices

Based on the normalized variance decomposition matrix, the core connectedness metrics are established:

The Total Connectedness Index (TCI), which captures the system-wide risk interdependence by calculating the proportion of cross-market spillovers relative to the total system variance, is expressed as follows:

(5) $$TCI\left(H\right)=\frac{\sum_{i\neq j}{\tilde{\theta }}_{ij}\left(H\right)}{\sum_{i,j}{\tilde{\theta }}_{ij}\left(H\right)}\times 100=\frac{\sum_{i\neq j}{\tilde{\theta }}_{ij}\left(H\right)}{N}\times 100$$

The directional spillover indices capture the risk received by market *i* from all other markets (FROM) and the risk transmitted by market *i* to all other markets (TO), formulated respectively as follows:

(6) $$FROM_{i\leftarrow \cdot }\left(H\right)=\sum_{j=1,j\neq i}^{N}{\tilde{\theta }}_{ij}\left(H\right)\times 100$$

(7) $$TO_{i\to \cdot }\left(H\right)=\sum_{j=1,j\neq i}^{N}{\tilde{\theta }}_{ji}\left(H\right)\times 100$$

The Net Spillover Index (NET) determines whether market *i* acts as a net transmitter or a net receiver of risk within the system:

(8) $$NET_{i}\left(H\right)=TO_{i\to \cdot }\left(H\right)-FROM_{i\leftarrow \cdot }\left(H\right)$$

A positive value ($NET_{i}>0$) implies that market *i* is a net risk transmitter, whereas a negative value ($NET_{i}<0$) indicates that it is a net risk receiver.

To capture the time-varying evolutionary features of risk connectedness, we implement a rolling-window estimation method with a window length of w=250 trading days (approximately one calendar year). Furthermore, to explore directional asymmetries in risk transmission, the underlying return series are decomposed into positive shocks ($r^{+}=\max\left(r,0\right)$) and negative shocks ($r^{-}=\min\left(r,0\right)$). This enables the separate construction of “good” and “bad” volatility spillover networks [49] to evaluate structural variations under different shock regimes.

### 3.2. Effective Transfer Entropy (ETE)

While the Diebold–Yilmaz framework provides a robust linear baseline, it remains constrained by the linear constraints of underlying VAR specifications and cannot capture non-linear dependencies across financial markets. To address this limitation, we introduce Effective Transfer Entropy (ETE) as a non-linear measure of directional information transmission. Developed within information theory by Schreiber [30], transfer entropy quantifies the predictive capacity of one time series over another by evaluating divergence in conditional probability distributions, rendering it intrinsically suitable for detecting non-linear directional information flows and predictive dependence.

#### 3.2.1. Fundamental Formulation of Transfer Entropy

Let *I* and *J* denote two discrete stationary random processes, and let $I_{t}^{\left(k\right)}=\left(I_{t},I_{t-1},\ldots ,I_{t-k+1}\right)$ and $J_{t}^{\left(l\right)}=\left(J_{t},J_{t-1},\ldots ,J_{t-l+1}\right)$ represent their respective histories. The transfer entropy from process *J* to process *I* is formally defined as follows:

(9) $$TE_{J\to I}\left(k,l\right)=\sum p\left(i_{t+1},i_{t}^{\left(k\right)},j_{t}^{\left(l\right)}\right)\log\frac{p(i_{t+1}|i_{t}^{\left(k\right)},j_{t}^{\left(l\right)})}{p(i_{t+1}|i_{t}^{\left(k\right)})}$$

where p(·) denotes the joint or conditional probability density functions. Transfer entropy effectively captures the extent to which the inclusion of *J*’s historical information reduces the uncertainty regarding the future states of *I*, over and above the history of *I* alone. A positive value ($TE_{J\to I}>0$) indicates directional information transmission from *J* to *I*. This formulation shares a conceptual alignment with generalized Granger causality but is freed from any assumption of linear structures.

Given that financial asset returns are continuously distributed while transfer entropy estimation operates on discrete state probabilities, we utilize a quantile-based discretization approach to symbolize the return sequences. Specifically, the sample observations are mapped into three discrete regimes—downward, stable, and upward—based on their empirical distribution. This symbolic transformation mitigates the distortions induced by extreme outliers and enhances the robustness of the resulting entropy estimates.

In this implementation, discretization into three regimes (downward, stable, upward) follows tercile quantile thresholds (33% and 67%) internally implemented within the RTransferEntropy package (R version 4.3.1; RTransferEntropy package version 0.2.2). The history-length parameters for past realizations are set to k=l=1. Statistical significance of each directional information flow is evaluated using the built-in bootstrap procedure with baseline B=100 shuffling replications. We further conduct sensitivity checks using alternative bootstrap replications B=80 and B=120. The magnitude of pairwise effective transfer entropy and the net information-flow ranking across markets remain qualitatively stable across these settings.

We emphasize that ETE quantifies directional predictive dependence rather than structural economic causality; a non-zero ETE value indicates that the past information of one series improves the prediction of another, but does not by itself identify a causal mechanism or rule out common exogenous driving factors.

#### 3.2.2. Small-Sample Bias Correction via Effective Transfer Entropy

In finite samples, transfer entropy estimates are highly prone to small-sample biases that generate artificial non-zero values. To isolate true information flows, we adopt the Effective Transfer Entropy (ETE) framework proposed by Marschinski and Kantz [31]:

(10) $$ETE_{J\to I}\left(k,l\right)=TE_{J\to I}\left(k,l\right)-TE_{J_{\mathrm{shuffled}}\to I}\left(k,l\right)$$

where $TE_{J_{\mathrm{shuffled}}\to I}$ is computed by randomly shuffling the elements of sequence *J*. This shuffling process effectively destroys the temporal dependence structure of *J* while strictly preserving its marginal distribution. The shuffling procedure is repeated across B=100 bootstrap replications, and the resulting average is utilized as the bias-correction term. By filtering out these spurious information flows, ETE effectively alleviates finite-sample distortion, where larger positive values indicate heightened intensities of non-linear information transmission.

### 3.3. Quantile Vector Autoregression (QVAR)

Spillover analyses evaluated at the conditional mean can systematically underestimate the intensity of risk interdependence under extreme market states.

This concern is not merely methodological but economic in nature. Systemic risk is, by definition, a tail phenomenon: the episodes that macroprudential regulators and investors care most about—severe market downturns, liquidity dry-ups, and crisis episodes—occur precisely in the lower and upper quantiles of the return distribution, not at the conditional mean. Moreover, the transmission mechanisms that dominate in such states are fundamentally different from those operating in normal markets: leverage constraints, margin calls, forced deleveraging, and loss-averse selling pressure activate asymmetrically during extreme moves, generating amplification and contagion patterns that a mean-based VAR, which imposes a single covariance structure across all states, cannot recover. A conditional-mean connectedness index therefore answers only the question “how do markets co-move on average?”, whereas the substantive question for cross-market risk analysis is “how do shocks propagate when one market is under severe stress?”

To capture tail risk propagation mechanisms across financial segments, this paper implements the Quantile Vector Autoregression (QVAR) model developed by Ando et al. [9], which characterizes the state-dependent risk connectedness structures across varying quantile levels.

#### 3.3.1. QVAR Model Specification

For a given quantile level τ∈(0,1), the QVAR(*p*) specification is formalized as follows:

(11) $$X_{t}=\mu \left(\tau \right)+\sum_{i=1}^{p}\Phi_{i}\left(\tau \right)X_{t-i}+\varepsilon_{t}\left(\tau \right)$$

where $X_{t}$ is the N×1 return vector, μ(τ) represents the quantile-dependent intercept vector, $\Phi_{i}\left(\tau \right)$ denotes the quantile-dependent coefficient matrices, and $\varepsilon_{t}\left(\tau \right)$ is the vector of error terms satisfying the conditional quantile restriction $Q_{\tau }\left(\varepsilon_{t}\left(\tau \right)|F_{t-1}\right)=0$. The parameters are estimated by minimizing the asymmetric objective function, often termed the check function [56]:

(12) $$\min\sum_{t=1}^{T}\rho_{\tau }\left(X_{t}-\mu \left(\tau \right)-\sum_{i=1}^{p}\Phi_{i}\left(\tau \right)X_{t-i}\right)$$

where $\rho_{\tau }\left(u\right)=u\left(\tau -I\left(u<0\right)\right)$ signifies the asymmetric absolute loss function.

#### 3.3.2. Spillover Indices Under the QVAR Framework

Following Ando et al. [9], we construct a quantile-specific moving average representation based on the estimated parameters:

(13) $$X_{t}=\sum_{j=0}^{\infty }A_{j}\left(\tau \right)\varepsilon_{t-j}\left(\tau \right)$$

This representation allows for the derivation of the generalized forecast error variance decomposition at specific quantile levels:

(14) $$\theta_{ij}^{\tau }\left(H\right)=\frac{\sigma_{jj}{\left(\tau \right)}^{-1}\sum_{h=0}^{H-1}{\left(e_{i}^{\prime }A_{h}\left(\tau \right)\Sigma \left(\tau \right)e_{j}\right)}^{2}}{\sum_{h=0}^{H-1}\left(e_{i}^{\prime }A_{h}\left(\tau \right)\Sigma \left(\tau \right)A_{h}{\left(\tau \right)}^{\prime }e_{i}\right)}$$

where Σ(τ) is the quasi-covariance matrix constructed from the residuals of the quantile estimations, and $\sigma_{jj}\left(\tau \right)$ represents its diagonal elements. This quasi-covariance structure scales the exogenous shocks at each specific quantile level, thereby guaranteeing the comparability of variance decompositions across different variables. On this basis, the quantile-dependent total connectedness index TCI(τ) and directional spillover indices are constructed.This paper focuses on three representative quantile regimes: extreme downward states (τ=0.05), median states (τ=0.50), and extreme upward states (τ=0.95), to comparatively map the transmission of risk under highly heterogeneous market environments.

### 3.4. Complex Network Analysis

To map the topological architecture of cross-market risk transmission, we construct a directed risk network based on the empirical ETE matrix. We define the node set as V={1,2,…,N}, representing the six core markets, where the weight of the directed edge $e_{ij}$ is determined by the value of $ETE_{j\to i}$. To isolate the primary pathways of risk transmission, we implement a disparity filtering and thresholding technique to extract the backbone network [48], retaining only the edges that satisfy:

(15) $$e_{ij}\in E_{\mathrm{backbone}}\Leftrightarrow ETE_{j\to i}>\delta$$

The filtering threshold δ is set at the 75th percentile of the full-sample ETE distribution (δ=0.00318). This setup aligns with standard network filtering practices in empirical finance to isolate high-intensity risk channels while eliminating weak background noise. To confirm the stability of our findings, robustness checks are executed across alternative threshold configurations. Within this extracted backbone network, the following topological metrics are evaluated:

1. Network Density: The ratio of the number of active edges to the maximum possible connections, measuring the overall cohesion of the system.
2. PageRank Centrality: A sophisticated centrality metric adapted for directed, weighted graphs that evaluates a node’s systemic importance by considering the weights of its incoming links and the centrality of its neighbors [46]. It is recursively defined as follows:
  (16) $$PR_{i}=\frac{1-d}{N}+d\sum_{j\in In(i)}\frac{w_{ji}}{\sum_{k\in Out(j)}w_{jk}}PR_{j}$$
  where d=0.85 is the standard damping factor, In(i) is the set of nodes pointing to market *i*, Out(j) is the set of nodes targeted by market *j*, and $w_{ji}$ represents the edge weight.
3. Net Flow: The difference between a node’s out-degree (sum of outgoing edge weights) and its in-degree (sum of incoming edge weights), quantifying whether a market serves as a net sender or a net recipient within information streams.

Although the Diebold–Yilmaz index, Effective Transfer Entropy, and the QVAR framework all assess interconnectedness, they isolate fundamentally distinct dimensions of risk transmission, and each addresses a blind spot left by the other two. The DY framework estimates linear volatility spillovers at the conditional mean. It tells us, on average and under a linear VAR structure, how shocks in one market propagate to others. Its two limitations are that it assumes a linear functional form and that it imposes a single covariance structure across all market states. ETE directly resolves the first limitation: by operating on conditional probability distributions without assuming linearity, it reveals directional nonlinear predictive dependence that a linear VAR would miss. However, ETE is estimated over the full sample and does not distinguish between normal and stress states, so it cannot tell us whether the information flow is stable or regime-dependent. QVAR directly resolves the second limitation: by estimating connectedness at different quantiles of the return distribution, it reveals how spillover intensity changes between median and tail states, even though it retains a linear quantile specification. Consequently, these three methods are highly complementary, together establishing a comprehensive identification framework for cross-market risk propagation mechanisms.

It should also be noted that the DY-based spillover index captures linear volatility connectedness at the conditional mean, whereas ETE characterizes higher-order nonlinear information flows. Market roles identified from these two frameworks may therefore differ without being contradictory.

## 4. Data Preprocessing and Statistical Characteristics

### 4.1. Data Description and Sample Period

This study selects six core markets in China as the objects of empirical investigation: the stock market (Stock), bond market (Debt), foreign exchange market (Forex), real estate market (Real Estate), energy market (Energy), and high-tech market (High-tech). Daily observations are constructed using representative indices or asset price series for each market to guarantee consistency and comparability across statistical dimensions. The representative proxy variables and corresponding data sources for the six markets are summarized in Table 1.

The high-tech market index is specifically constructed as a Paasche index based on a representative basket of component stocks. This specification offers the distinct advantage of capturing the price dynamics induced by industrial structural adjustments and shifting sector weights over time. Formally, the Paasche price index aggregates constituent closing prices weighted by base-period trading volume:

(17) $$P_{t}^{P}=\frac{\sum_{i\in \Omega }p_{i,t}q_{i,0}}{\sum_{i\in \Omega }p_{i,0}q_{i,0}}$$

where Ω denotes the pool of constituent sub-sector indices; $p_{i,t}$ is the closing value of sub-sector index *i* on day *t*; $p_{i,0}$ and $q_{i,0}$ are the base-period index value and base-period trading volume, respectively, with the base period corresponding to the earliest date in our sample.

The constituent pool Ω comprises five representative high-tech sub-sector indices: the Wind Semiconductor Index (882121.WI), the Wind Aerospace & Military Industry Index (886015.WI), the CITIC Communications Index (CI005026.WI), the CSI Computer Index, and the CSRC Pharmaceutical Manufacturing Index. These five indices collectively cover the core high-tech value chains in China: semiconductors, defense electronics, telecommunications equipment, software and information services, and biopharmaceutical manufacturing.

This constructed high-tech index serves as a core input to the DY-Yilmaz spillover, effective transfer entropy, and QVAR tail-risk connectedness analyses, so that several central empirical conclusions rely on this indicator.

#### 4.1.1. Construction of Return Sequences

To satisfy the stationarity requirements of time-series econometric modeling, raw price sequences $P_{i,t}$ are transformed into logarithmic returns as follows:

(18) $$r_{i,t}=100\times \left(\ln P_{i,t}-\ln P_{i,t-1}\right)$$

This formulation provides time additivity and effectively characterizes continuously compounded return features. Compared with simple net returns, logarithmic returns exhibit superior statistical properties in high-frequency financial econometrics.

#### 4.1.2. Data Cleaning and Missing Value Processing

Given that trading calendars vary across distinct asset segments, we implement a maximum common trading day filtering approach to align the asynchronous observations. Although this procedure enhances sample alignment, it may introduce synchronous trading biases; thus, its empirical implications are evaluated via robustness checks in subsequent sections. For missing observations, a localized linear interpolation is applied to single-period missing entries, while consecutive missing intervals are discarded to mitigate the distortions from non-trading factors.

Concurrently, the return sequences are winsorized at the 1–99% quantile levels. We emphasize that this procedure does not eliminate tail information: the winsorization bounds correspond to the first and ninety-ninth percentiles, whereas the QVAR tail analysis focuses on the fifth and ninety-fifth percentiles. The purpose of winsorization is to prevent a small number of data-entry errors or microstructure anomalies from distorting the covariance matrix underlying the DY generalized forecast error variance decomposition, which is sensitive to extreme single observations. Quantile regression itself, including the QVAR estimator, is robust to outlying observations because it models conditional quantiles rather than conditional means.

The comprehensive sample period spans from 2004 to 2024, encapsulating multiple major macroeconomic and financial shocks, such as the Global Financial Crisis, the COVID-19 pandemic, and domestic real estate adjustment cycles, thereby ensuring strong structural heterogeneity and shock representativeness.

To verify empirically that these preprocessing choices do not drive the results, we re-estimated the full model under two alternative schemes. In the first scheme, we removed winsorization entirely while retaining the common-trading-day filter. In the second scheme, we retained all calendar dates and filled missing observations by linear interpolation before winsorization. Under the first scheme, the DY total connectedness index changes only marginally (36.73% versus 36.33% in the baseline), the net spillover positions of all six markets remain virtually identical (e.g., stock net transmitter +15.32 versus +15.37; high-tech net receiver −11.46 versus −11.17), the ETE net information-flow ranking is unchanged, and the QVAR tail connectedness values at τ=0.05/0.50/0.95 are 73.88%/36.26%/73.81%, closely matching the baseline (75.34%/35.87%/75.28%). Under the second scheme, interpolation lowers the total connectedness to 27.15% and reorders some ETE flows, which is expected because aligning markets on non-synchronous trading days introduces artificial co-movement; nevertheless, the U-shaped QVAR tail structure and the dominant roles of the stock market (net transmitter) and high-tech sector (net receiver) are preserved. These checks confirm that the baseline preprocessing choices are not driving the headline conclusions.

### 4.2. Descriptive Statistical Analysis

Table 2 reports the descriptive statistics for the return sequences of the six markets.

In terms of conditional means, the average daily returns across all markets hover close to zero, aligning with the stylized facts of financial asset returns. The high-tech market exhibits the highest mean return (0.0444), indicating strong growth momentum and capital expansion capacity over the sample period.

Regarding volatility (standard deviation), the high-tech market displays the highest volatility level (2.9433), followed closely by the real estate (2.0031) and energy (1.8446) markets, revealing intensive price fluctuations and heightened risk exposure within these segments. Conversely, the bond market records the lowest volatility (0.0658), confirming its inherent stability and safe-haven properties.

The skewness and kurtosis metrics collectively indicate pronounced leptokurtic and fat-tailed distributions. Significant negative skewness is concentrated in the stock, real estate, and energy markets, implying that extreme downward shocks are more severe within these areas. Moreover, all kurtosis values markedly exceed the Gaussian benchmark of 3, underscoring severe systemic tail risks.

Finally, the Jarque–Bera test statistics demonstrate that, with the exception of the high-tech market, all asset returns reject the null hypothesis of normality at the 1% significance level, confirming the ubiquitous non-Gaussian behavior of domestic financial returns.

### 4.3. Stationarity and ARCH Effect Tests

To guarantee the validity of parameter estimations in our VAR-based frameworks, we perform the Augmented Dickey–Fuller (ADF) unit root test on the six return series. Table 3 presents the test results. The ADF statistics for all series reject the null hypothesis of a unit root at the 1% significance level, establishing that all return sequences are integrated of order zero, I(0), which satisfies the baseline prerequisite for subsequent VAR modeling.

Furthermore, we implement the ARCH-LM test (with a lag order of 12) to inspect the presence of conditional heteroscedasticity. As reported in Table 4, the ARCH test statistics are highly significant at the 1% level across all six markets, confirming the ubiquitous existence of ARCH effects. This finding indicates that domestic asset returns display prominent volatility clustering—large price variations tend to be followed by large variations, and small variations by small variations.

These properties document the time-varying nature of financial market uncertainty, validating the statistical foundations for our subsequent spillover indexing and volatility modeling.

### 4.4. Cross-Market Correlation Analysis

Table 5 presents the unconditional correlation matrix among the six asset return sequences.

The unconditional parameters reveal that the strongest correlation occurs between the stock and energy markets (0.8325), followed closely by the stock and real estate alliance (0.7800). These intense linkages point toward a pronounced co-movement structure that is highly susceptible to risk resonance. In contrast, the bond market maintains weak negative correlations with all other risk assets, validating its role as a traditional defensive hedging asset. Because these static measures capture neither directional causality nor information transmission, they necessitate the dynamic spillover indexing and information-theoretic formulations executed below. Overall, the synchronized linkages across Chinese financial markets establish a robust empirical baseline for mapping cross-market risk networks.

### 4.5. Descriptive Temporal Characteristics

**Figure 1:**
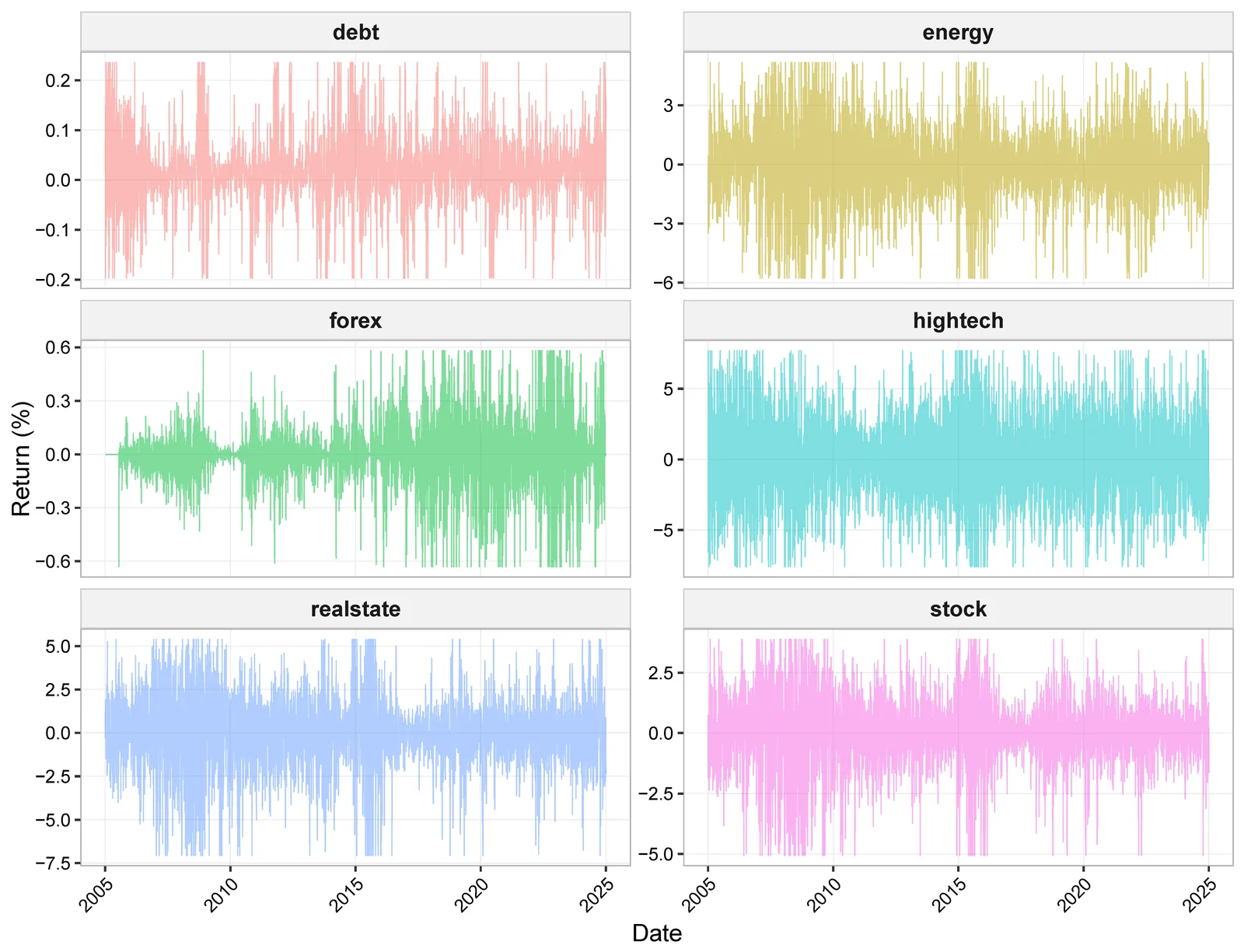
Time-series trajectories of returns across the six core markets.

Figure 1 maps the time-series evolution of the six return sequences. The plots visually confirm that market volatility is highly time-varying and exhibits intensive clustering.

The high-tech segment displays the widest variation amplitudes, while the bond market presents long-term low-volatility patterns. During major structural disruptions—such as the Global Financial Crisis of 2008, the domestic equity market turbulence of 2015, and the COVID-19 shock of 2020—the stock, real estate, and energy markets exhibit highly synchronized expansions in volatility, whereas the bond market offers stable counter-cyclical buffering. These observations imply that cross-market risk coupling amplifies significantly under extreme stress conditions, providing an intuitive justification for dynamic connectedness modeling.

## 5. Static and Dynamic Characteristics of Risk Spillovers

This chapter investigates the risk transmission architecture across six core Chinese markets (stock, bond, foreign exchange, real estate, energy, and high-tech) from both static and dynamic perspectives based on the spillover index framework proposed by Diebold and Yilmaz (2012) [20]. The static analysis characterizes the average interconnectedness structure across the full sample period, whereas the dynamic rolling-window analysis identifies the time-varying properties of risk spillovers and their potential state-dependent transitions.

### 5.1. Static Spillover Analysis

Table 6 reports the static risk spillover matrix calculated via the Generalized Forecast Error Variance Decomposition (GFEVD). The Total Connectedness Index (TCI) of the system stands at approximately 36.5%, indicating that roughly one-third of the forecast error variance can be explained by cross-market shocks. This underscores a significant systemic co-movement structure among asset markets rather than mutually independent risk evolution processes.

Regarding the net spillover positions of individual markets, the stock market (NET = 15.54) emerges as the predominant net risk transmitter, with its outward transmission intensity (TO = 76.92) significantly exceeding its inward reception intensity (FROM = 61.37). This suggests that the stock market serves as a stable origin for risk propagation within the system. Conversely, the bond market (NET = −2.36) exhibits a small negative net spillover position in the full-sample static estimation, with very low cross-market spillover magnitudes overall. Its dynamic rolling-window net spillover average turns more negative (−5.75), revealing that the bond market acts as a prominent risk-absorbing safe-haven asset especially during crisis episodes.

The foreign exchange market (NET = −2.39) and the energy market (NET = −0.03) operate as mild net risk receivers in the full-sample static estimation, indicating that they are only modestly affected by shocks from other asset classes during the sample period. The high-tech market (NET = −11.13) acts as a strong net receiver from a full-sample perspective, with its inward reception intensity substantially exceeding its outward transmission, revealing its pronounced vulnerability to cross-market risk shocks.

**Figure 2:**
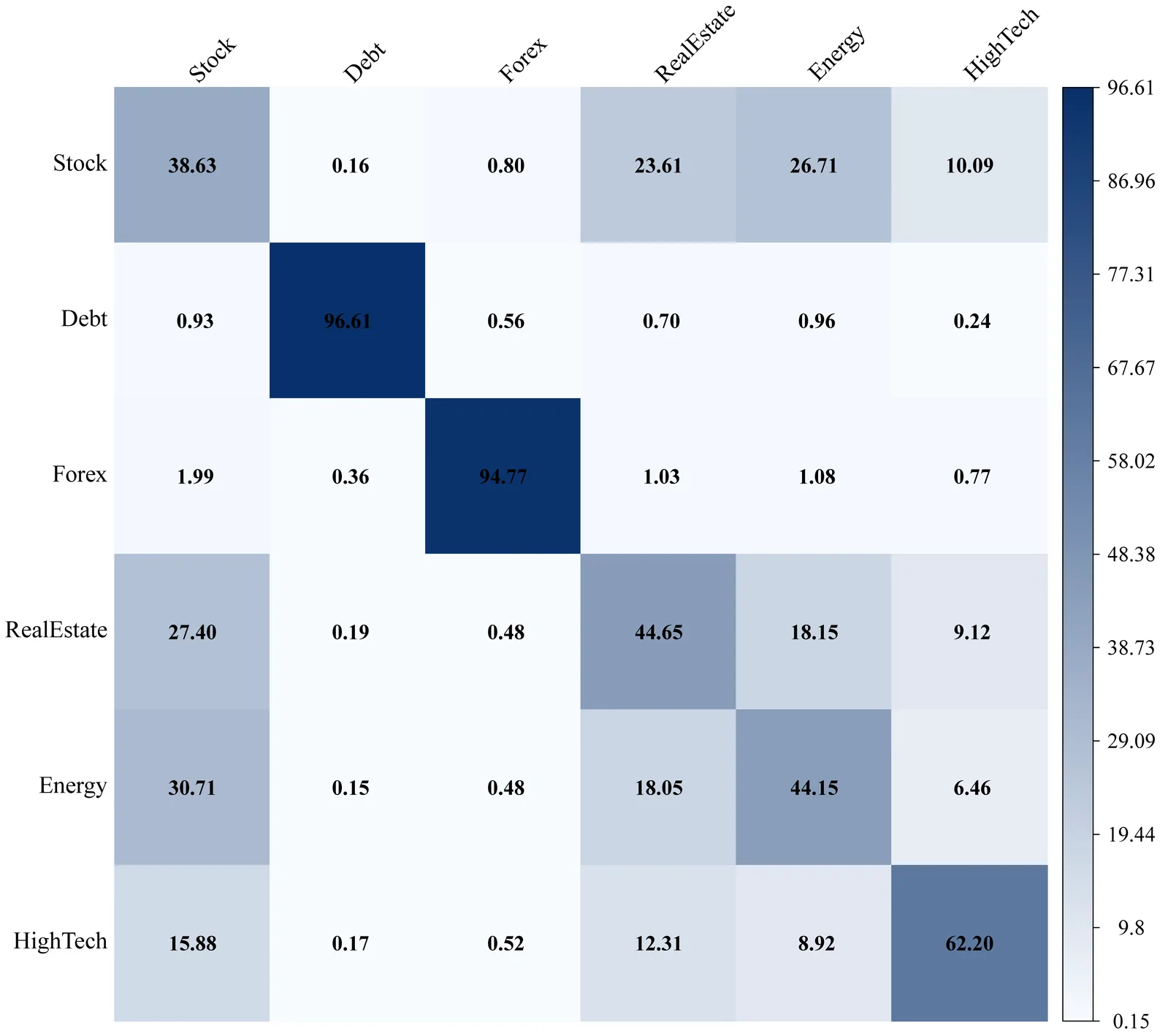
Heatmap of static risk spillovers.

Figure 2 further illustrates the intensity distribution of pairwise spillovers reported in Table 6. The results indicate that directional spillovers are highly pronounced in the stock–energy (30.71%), stock–real estate (27.40%), and real estate–stock (23.61%) directions, demonstrating a significant co-movement pattern among traditional risk assets characterized by cross-industry contagion.

### 5.2. Dynamic Total Spillover Analysis

Given that the interconnectedness of financial markets may adjust alongside variations in the macroeconomic environment, this study further employs a rolling-window methodology (with a window length of 250 trading days) to calculate the dynamic Total Connectedness Index (TCI), thereby capturing the time-varying features of systemic connectedness.

**Figure 3:**
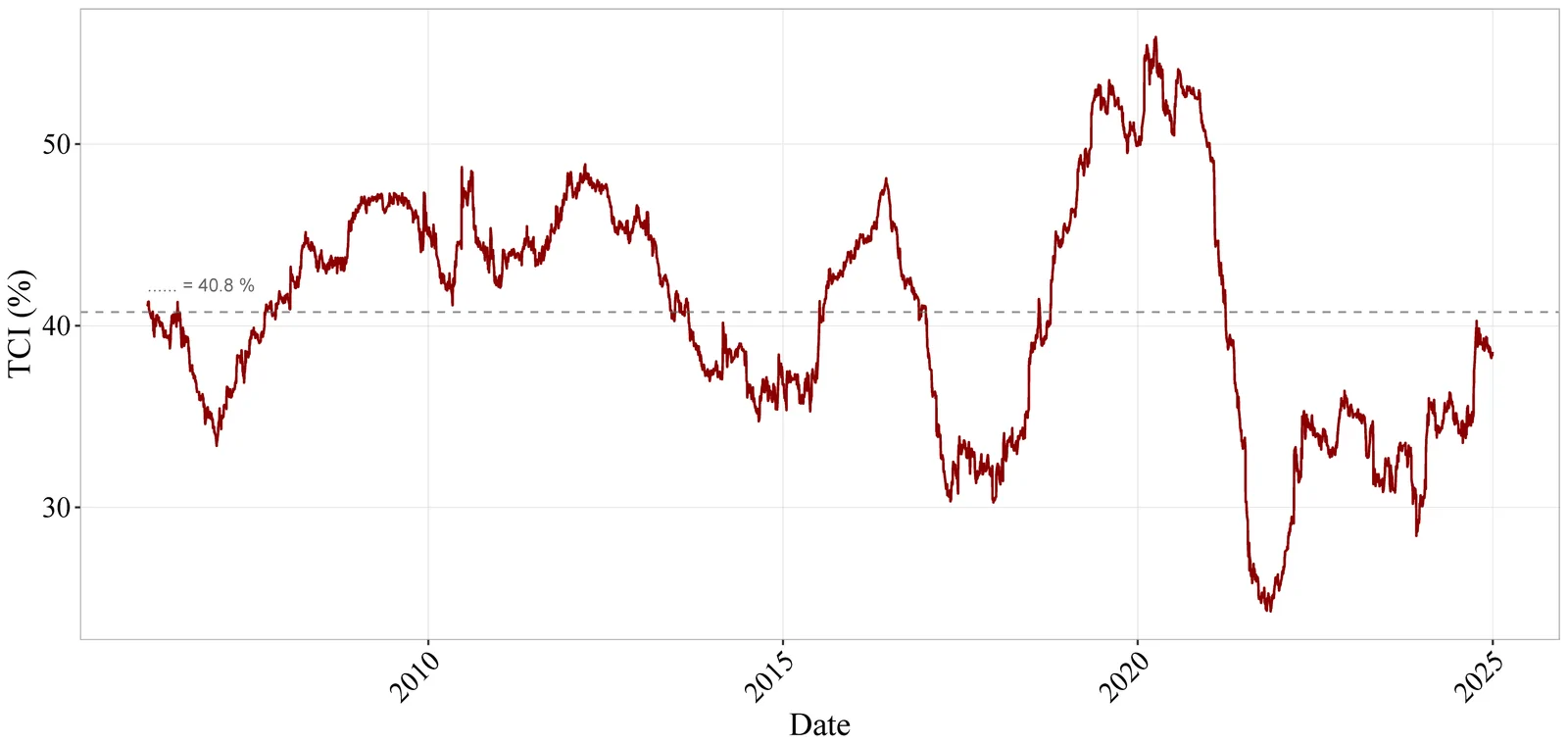
Dynamic evolution trajectory of the systemic Total Connectedness Index (TCI).

As depicted in Figure 3, the TCI exhibits prominent time-varying characteristics and episodic fluctuations throughout the sample period. Overall, the systemic risk connectedness remains sustained at a high level over the long term, indicating robust risk transmission channels among the six Chinese markets.

From the perspective of temporal evolution, the TCI escalated significantly during the 2008 Global Financial Crisis, implying that external financial shocks substantially intensified cross-market risk co-movements. Subsequently, during the 2015 domestic equity market turbulence, the TCI experienced another rapid surge and reached its sample-period peak around 2019–2020. This suggests that cross-market risk propagation channels are notably reinforced under major uncertainty shocks, thereby driving up systemic risk connectedness.

Notably, after 2020, the TCI underwent a rapid deceleration, dropping to a sample-period low around 2021. This reflects a mitigation of cross-market risk coupling following macroeconomic policy interventions and the gradual release of market stress. Thereafter, the TCI demonstrated a volatile recovery; although systemic connectedness experienced a rebound, it did not return to its previous historical peaks.

Statistically, the dynamic TCI averages approximately 40.8%, demonstrating that, on average, more than 40% of the forecast error variance originates from cross-market risk shocks, further confirming the robust systemic connectedness and risk transmission attributes of Chinese financial markets.

### 5.3. Dynamic Net Spillover Analysis

**Figure 4:**
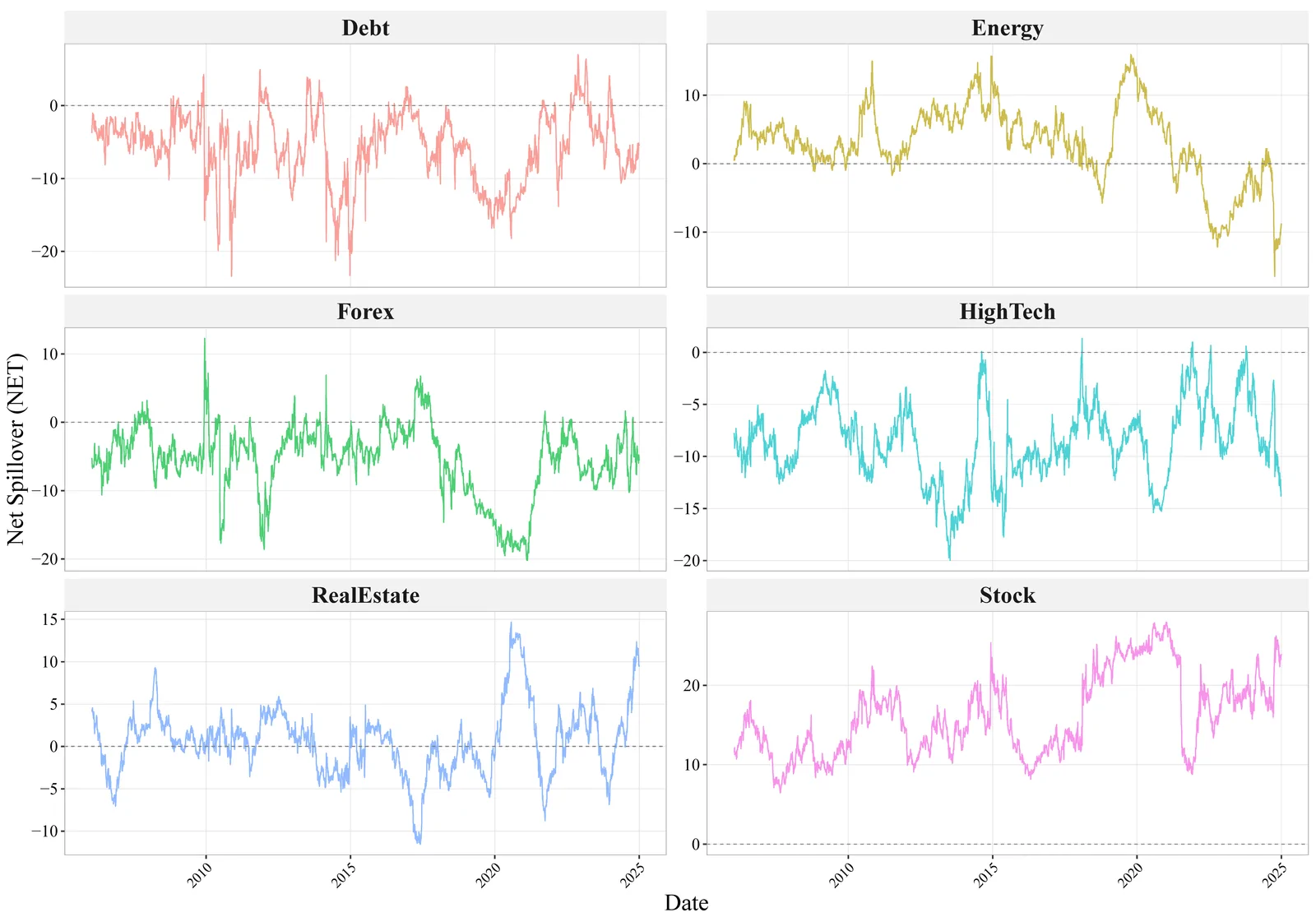
Dynamic evolution of net risk spillovers (NET) across the six core markets.

To further delineate the behavioral variations in individual markets under different market states, we compute the dynamic net spillover index for each market, the evolution pathways of which are illustrated in Figure 4.

According to the dynamic conditional means (Table 7), the stock market consistently maintains a positive net spillover position (16.10), confirming its function as a relatively stable risk transmitter across diverse market conditions. The dynamic NET average for the bond market drops further to −5.75 compared to its static counterpart, implying that its safe-haven performance becomes more pronounced during crisis periods.

The energy market switches from a nearly neutral position under full-sample static estimation to a positive net transmission stance in dynamic rolling-window estimates (3.12), indicating that its risk role possesses a pronounced degree of state dependence across the time dimension. The high-tech market exhibits a dynamic average of −8.59, but displays a clear negative-to-positive reversal in the latter phase, which suggests that its structural role in risk propagation is undergoing a fundamental transformation.

The real estate market remains close to an equilibrium state on average (0.57) while displaying prominent episodic variations.

## 6. Time-Varying Properties and Asymmetric Characteristics of Risk Spillovers

Building upon the identification of the baseline roles of individual markets, this section further explores whether significant structural discrepancies exist within the risk transmission mechanisms when financial markets encounter shocks of varying desires (i.e., positive versus negative shocks). By decomposing the return sequences into positive shocks ($r^{+}$) and negative shocks ($r^{-}$), we systematically examine the asymmetric characteristics of risk spillovers and their topological evolutionary patterns within complex financial networks.

### 6.1. Static Positive and Negative Spillover Decomposition

Table 8 presents the static net risk spillover indices derived from the full-sample decomposition. The empirical results reveal distinct “role transitions” within Chinese financial markets under different types of shocks. The stock market consistently maintains robust net risk-transmitting capacity under both configurations, establishing its stability as a primary source of systemic risk; conversely, the high-tech market exhibits substantially higher risk reception intensity under negative shocks than under positive ones, demonstrating its heightened sensitivity to systemic panic shocks.

Based on the asymmetric impact index (Diff), the real estate market exhibits the most pronounced asymmetric features. Under positive shocks, the real estate market operates as a significant risk receiver; however, under negative shocks, its net spillover rapidly shifts into positive territory. This pattern indicates that the real estate market possesses a noticeable pro-cyclical risk amplification effect, making it highly susceptible to transforming from a risk absorber into a net risk transmitter during market downturns, thereby serving as a critical channel for systemic risk propagation. Similarly, the energy market displays a moderate degree of asymmetry, implying that the contagion intensity induced by downward energy price shocks surpasses the outward spillover effects generated by upward price movements.

### 6.2. Dynamic Asymmetric Spillovers

**Figure 5:**
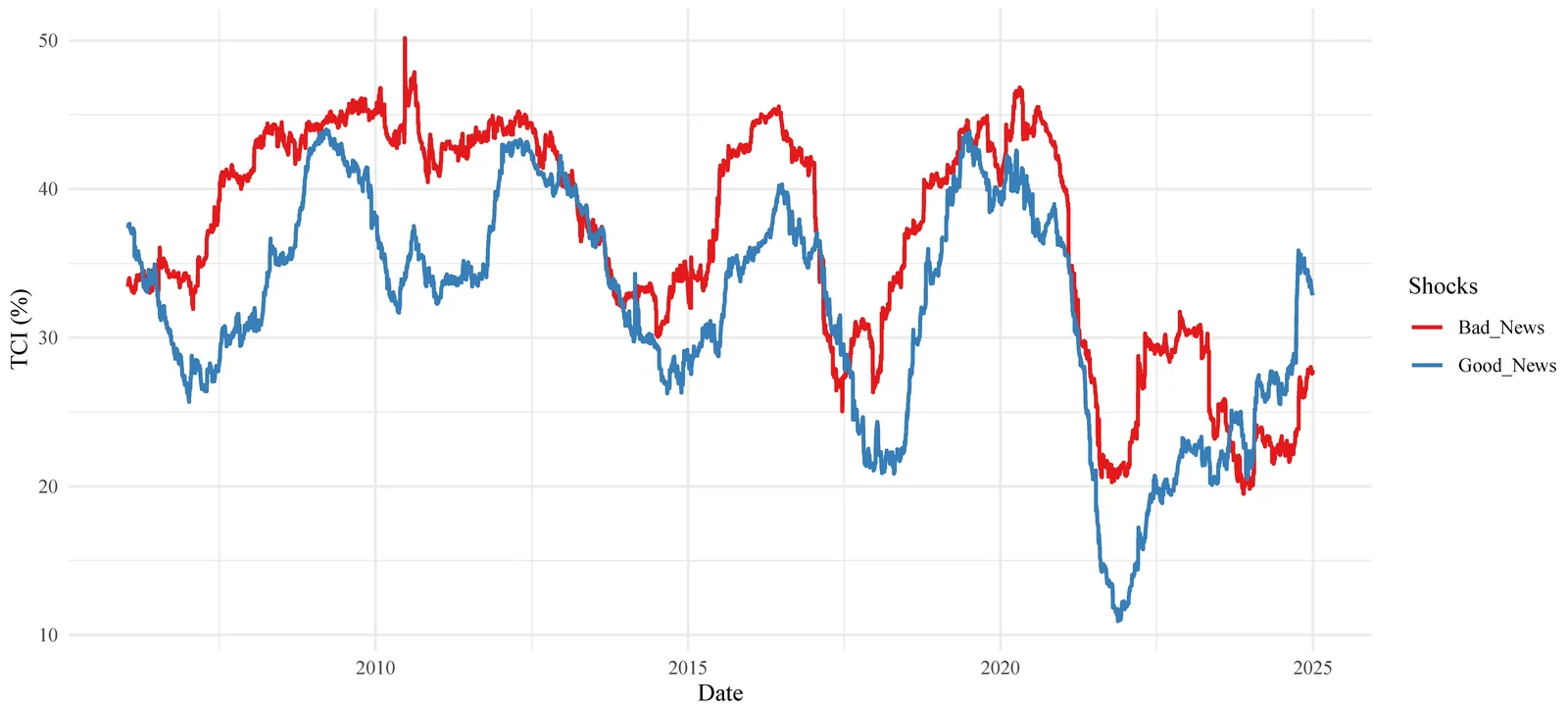
Comparison of dynamic Total Connectedness Indices: positive shocks vs. negative shocks.

To capture the evolutionary trajectories of asymmetry over the temporal dimension, we employ a rolling-window methodology to compute the dynamic positive and negative total connectedness indices. As illustrated in Figure 5, the total spillovers corresponding to negative shocks consistently surpass those of positive shocks across most of the sample period. This finding validates the presence of a typical “bad news amplification effect” in financial markets, where the intensity of cross-market risk coupling during market contractions is significantly higher than during expansionary phases. Notably, during extreme periods such as the 2015 domestic equity market turbulence and the 2020 COVID-19 pandemic shock, the divergence between positive and negative spillovers expanded substantially. This indicates that extreme stress shocks not only elevate overall systemic connectedness but also significantly accelerate the marginal propagation efficiency of negative information.

### 6.3. Topological Characteristics of Risk Spillover Networks

To further delineate the transmission features under shocks of varying natures from a complex network perspective, we construct directional positive shock networks ($r^{+}$) and negative shock networks ($r^{-}$), and compute their corresponding global network metrics. The results are summarized in Table 9.

The empirical findings indicate that the risk propagation networks across the six core Chinese markets exhibit a pattern characterized by “structural stability paired with intensity asymmetry” under shifting shock environments.

First, network metrics such as network density, active edges, and the clustering coefficient remain identical across both network structures. This implies that the cross-market risk transmission paths possess strong structural robustness, demonstrating that variations in shock regimes do not alter the baseline topological skeleton of risk propagation.

However, risk transmission efficiency and intensity enhance substantially under negative shock conditions. Specifically, the average weight of the negative shock network increases by 20.44% compared to its positive counterpart, revealing that cross-market linkages intensify markedly during market downturns, whereby an idiosyncratic shock in a single segment can more easily propagate through established network pathways.

Concurrently, the average path length decreases by 21.86%, indicating that under negative shocks, risk information transmits rapidly via shorter channels across asset classes. Consequently, the financial system exhibits tighter coupling and higher information diffusion efficiency under adverse conditions.

In conclusion, Chinese financial markets are more susceptible to developing a “high-intensity, high-velocity” risk resonance mechanism under negative shocks. This implies that although the baseline network topology remains invariant, the nature of the shock significantly alters transmission efficiency, thereby accelerating the accumulation and propagation of systemic risks.

## 7. Nonlinear and Tail Risk Analysis

### 7.1. Nonlinear Directional Information Flows

Following the systemic spillover analysis embedded within the linear framework, this study introduces Effective Transfer Entropy (ETE) and Quantile Vector Autoregression (QVAR) models to capture the deep interconnectedness features of the financial system across the dimensions of nonlinear dependency structures and tail risk transmission.

Because conventional linear models are intrinsically constrained when characterizing asymmetries, state dependence, and higher-order moment interactions within asset return sequences, transfer entropy offers a robust nonparametric solution grounded in information theory. By executing directional measurements of shifts in conditional probability distributions, this methodology effectively identifies the trajectories and intensities of nonlinear information flows across distinct asset markets.

Before interpreting the estimates, we clarify that ETE values report predictive information transfer; they do not constitute structural causal evidence. Accordingly, the economic interpretations below are framed as associations consistent with proposed transmission channels, rather than identified causal effects.

According to the ETE empirical results summarized in Table 10, the information flows within the Chinese financial system manifest a pronounced directional asymmetry. Across the full sample period, the real estate market operates as a primary information source, with its directional transmission intensities to the high-tech sector (0.0055) and the stock market (0.0042) ranking among the largest pairwise directional information flows within the parameter matrix.

This empirical finding is consistent with the interpretation that the real estate market functions as a critical nexus within the Chinese financial architecture. Its price fluctuations contain localized sector-specific news and exhibit strong nonlinear predictive dependence regarding asset classes tied closely to the real economy and the broader capital market. We caution that this predictive dependence may reflect common exposure to macroeconomic fundamentals rather than a direct causal channel.

Concurrently, the high-tech segment and the stock market display a highly symmetric information feedback structure (with bidirectional ETE parameters both standing at 0.0031). This bidirectional predictive dependence is consistent with a stable, nonlinear interaction between the asset pricing dynamics associated with technological innovation and broader aggregate market expectations, though it does not by itself establish that technological innovation causally drives stock-market movements.

### 7.2. Evolutionary Characteristics of Tail Risk Spillovers

To further investigate the dynamic evolutionary mechanisms of asset markets under extreme conditions, this study utilizes a QVAR framework to construct the Total Connectedness Index (TCI) across conditional quantiles. This specification delineates the tail risk coupling configurations under extreme downward (τ=0.05) and extreme upward (τ=0.95) regimes.

**Figure 6:**
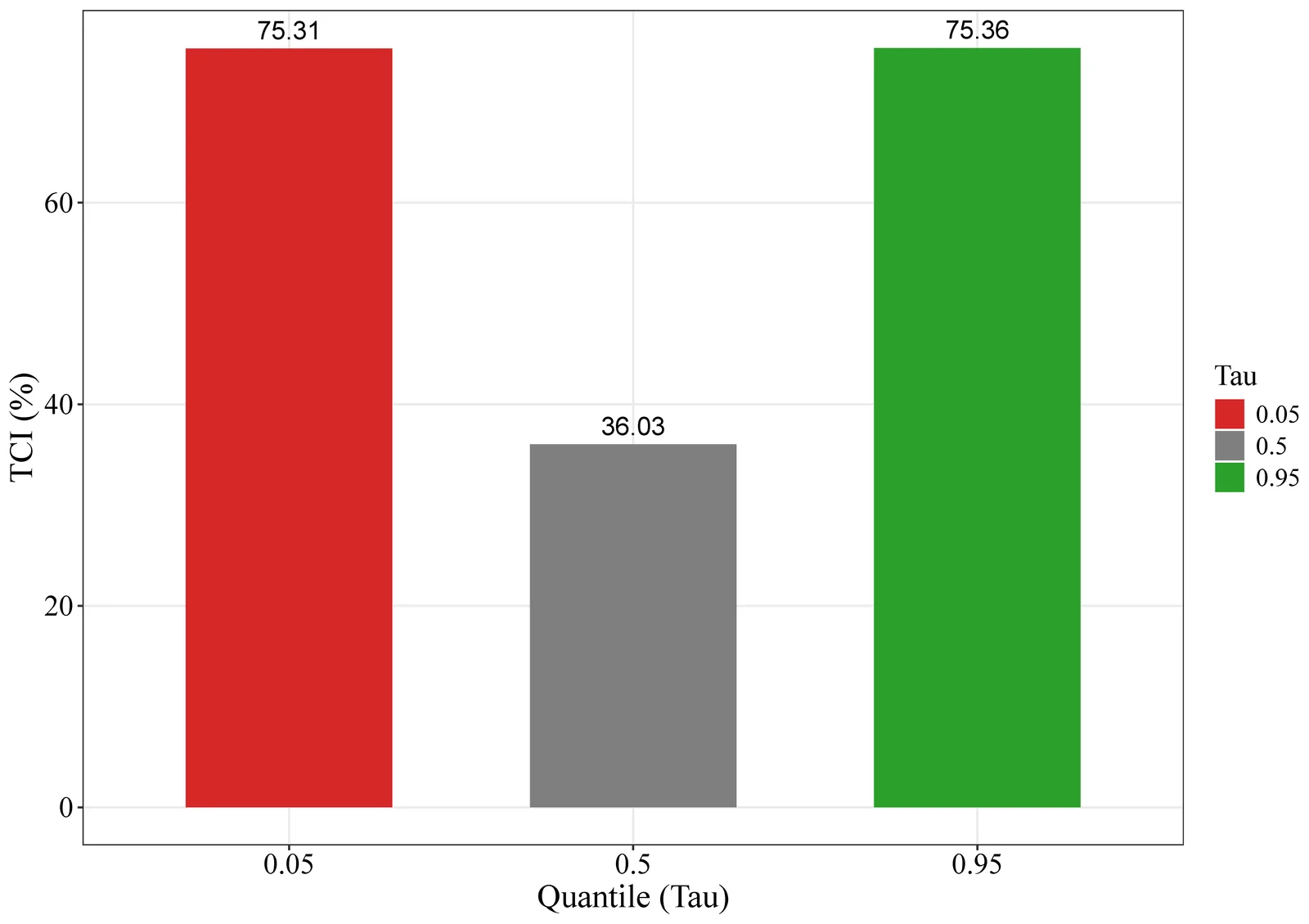
Distribution of the Total Connectedness Index (TCI) across conditional quantiles.

Figure 6 tracks the dynamic evolutionary paths of the TCI across different quantile levels. The systemic connectedness intensity reveals a distinct “U-shaped” distribution pattern. At the median state (τ=0.50), the total spillover level centers at approximately 36.03%, demonstrating that within normal market environments, a certain degree of risk diversification and buffering capacity persists among sub-markets. However, as the conditional quantiles expand toward the extreme tails, the connectedness parameters increase substantially, exceeding 75% under both extreme downward (τ=0.05) and extreme upward (τ=0.95) states. This indicates that regardless of whether the system experiences extreme panic or excessive optimism, cross-market risk couplings intensify, thereby impeding the endogenous risk diversification functions of the financial system.

In summary, the combined results from the nonlinear and tail risk investigations indicate that the information-leading paths of the real estate and high-tech sectors identified via transfer entropy constitute critical channels for extreme risk propagation. This transition sequence—moving from localized nonlinear dependencies to systemic tail risk resonances—reveals the highly coupled dynamic structural properties of Chinese financial markets under extreme conditions, establishing an integrative empirical baseline of information theory and econometrics to understand cross-market risk diffusion.

## 8. Topological Evolution of Risk Transmission Networks

### 8.1. Construction and Topological Properties of the Risk Transmission Backbone Network

**Figure 7:**
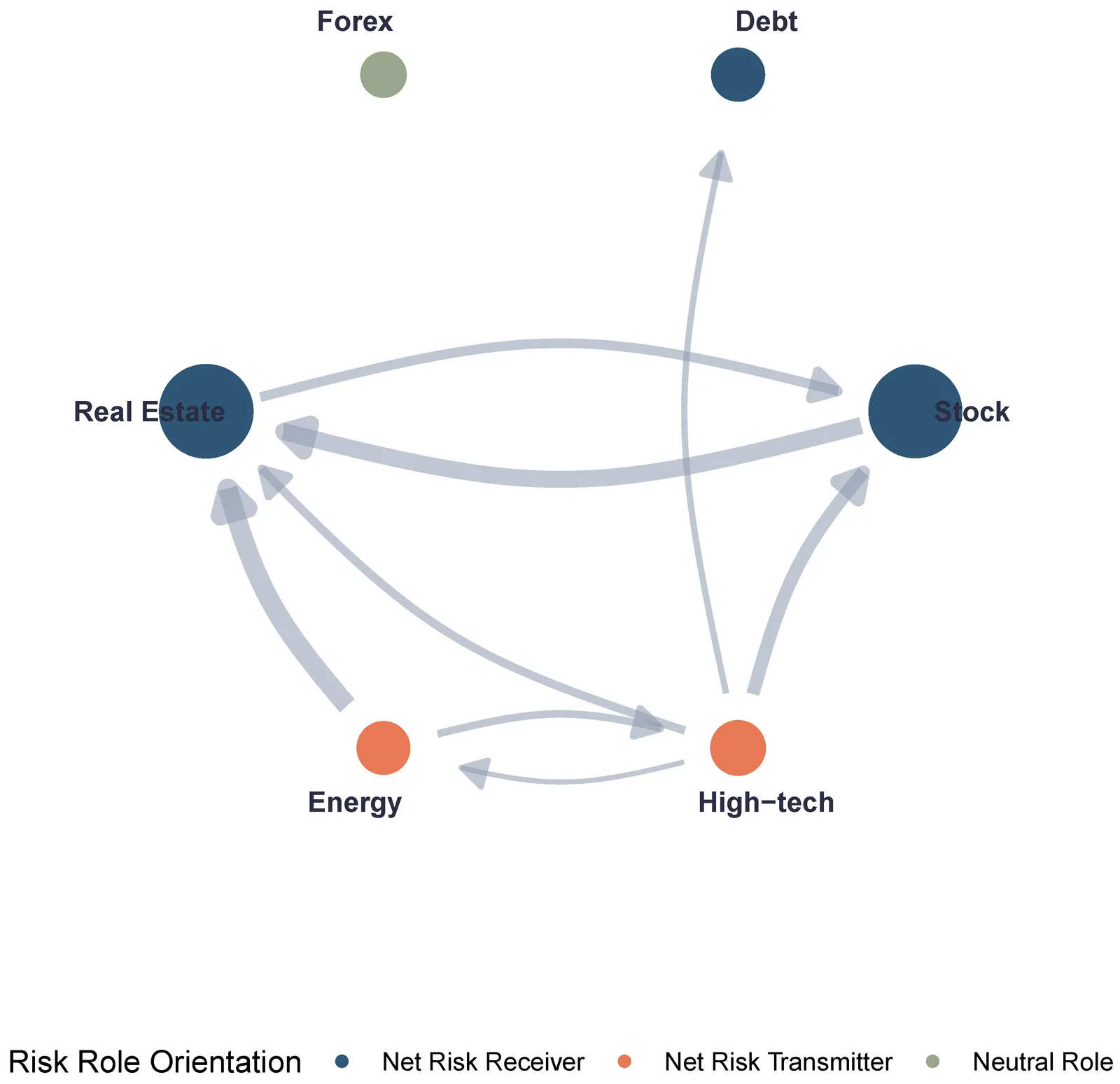
Structure of the Risk Transmission Backbone Network.

To extract statistically significant core risk transmission paths from complex nonlinear networks, this study applies a threshold truncation filtering technique based on the ETE matrix. By setting the 75th percentile of the ETE values as the structural cut-off (δ=0.00318), we construct a financial risk transmission backbone network. This procedure filters out weak linkages and stochastic noise, thereby highlighting the dominant high-intensity risk propagation channels. As illustrated in Figure 7, the backbone network presents distinct asymmetric and hierarchical properties.

In conjunction with the centrality metrics reported in Table 11, we identify the functional role orientations of individual asset classes within the network structure.

First, the high-tech sector exhibits considerable information spillover capacity and risk-leading features, with its net flow parameters reaching 0.0118, demonstrating a sustained net transmitting attribute within the nonlinear framework. This implies that over the sample period, nonlinear fluctuations within the high-tech sector maintain a leading character and generate expectations-guiding effects across other segments via information spillover channels.

Second, the real estate market and the stock market operate as net risk-receiving nodes. Notably, the real estate market registers a dominant PageRank centrality of 0.4319, confirming its role as a key channel within the risk propagation grid. This configuration indicates that the real estate sector absorbs external shocks originating from the high-tech and energy segments while simultaneously fulfilling an intermediary function of risk concentration and redistribution within the global network topology.

### 8.2. Event-Driven Dynamic Network Evolution

To capture the time-varying evolution of the risk transmission architecture, this study adopts a rolling-window methodology (with a window length of 250 trading days) to trace the dynamic trajectories of network density, marking major systemic risk shocks across the sample timeline.

**Figure 8:**
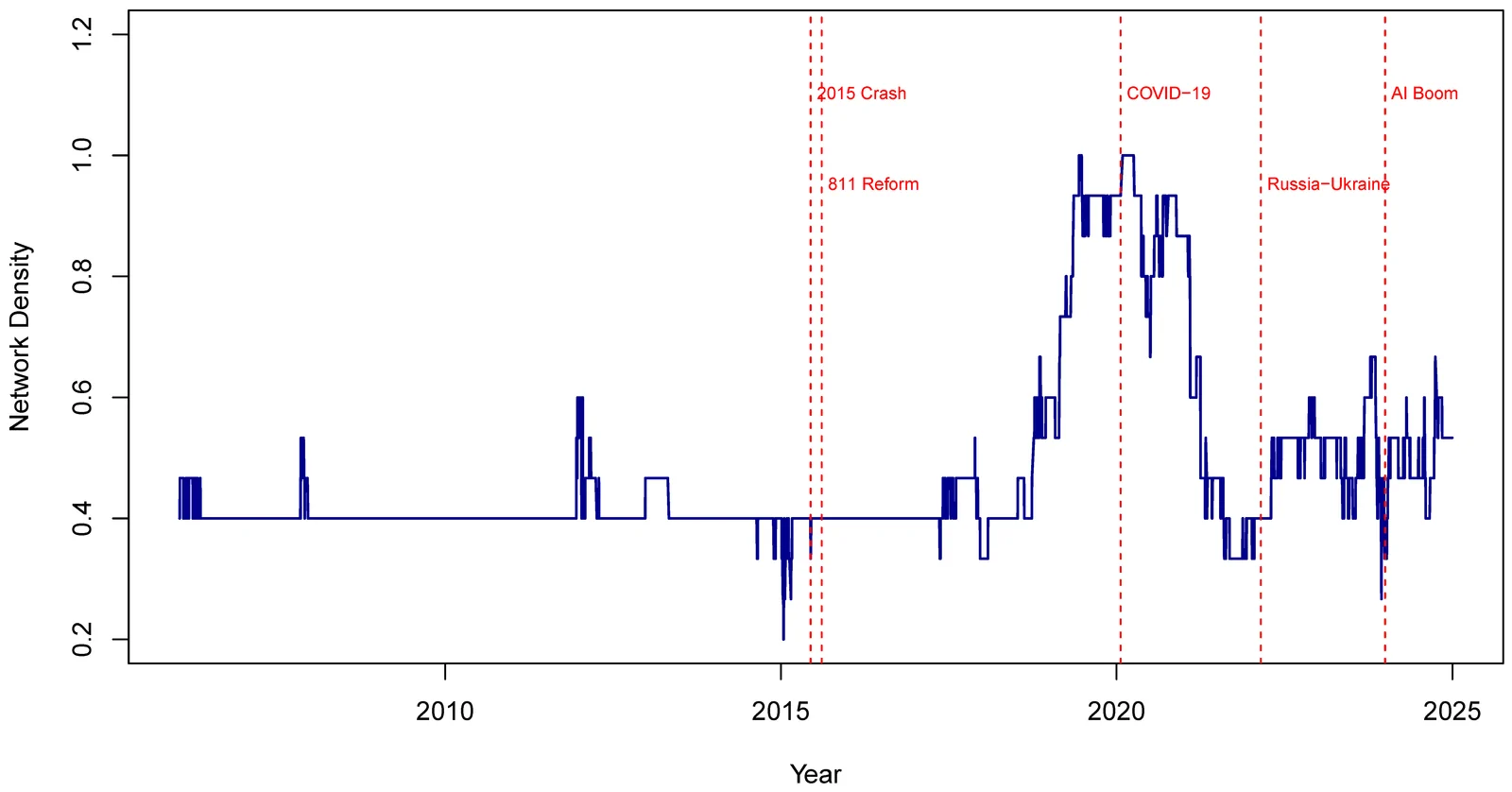
Time-varying Evolution of Network Density under Major Events.

As illustrated in Figure 8, the network density exhibits prominent pulse-like, time-varying evolutionary characteristics. During the 2015 Chinese stock market crash (2015 Crash) and the subsequent “811 Renminbi Exchange Rate Reform” (811 Reform), the network density did not undergo the conventional unorderly expansion typical of contagion phases; instead, it contracted sharply to a historical low of approximately 0.2. This phenomenon indicates that during such endogenous localized panics, non-linear information flows across markets are heavily impeded by a “circuit-breaker effect” induced by large-scale trading suspensions or extreme liquidity depletion, resulting in a temporary structural decoupling.

Conversely, under the exogenous macro-shock of the 2020 COVID-19 pandemic, the network density surged rapidly and remained sustained at a high level above 0.8 for a prolonged period, occasionally approaching the boundary of full coupling at 1.0. This stark contrast powerfully demonstrates that under systemic, extreme shock conditions, the originally decentralized risk structures of financial markets rapidly converge toward a highly coupled state, triggering a systemic “system-wide resonance” phenomenon. Furthermore, the 2022 geopolitical conflict (Russia–Ukraine) and the phased expansion of the Artificial Intelligence industry cycle since 2024 (AI Boom) have also induced stepwise increases in network density. This further underscores that geopolitical shocks and technological cycle evolutions, acting as novel non-linear driving forces, are profoundly reshaping the transmission density of cross-market risks.

### 8.3. Robustness Checks

To mitigate the subjectivity associated with the ETE backbone threshold selection, we perform a sensitivity analysis. By tracking network properties across the 70th, 75th, and 80th percentile specifications, we evaluate the structural robustness of our empirical outputs across varying degrees of network sparsity.

As reported in Table 12, as the percentile cut-off increases from 70% to 80%, the active edge count contracts from 9 to 6, filtering out extraneous paths to isolate the core channels. Across all configurations, the real estate market consistently maintains the highest PageRank centrality, while the high-tech sector remains the primary origin of net risk transmissions. These results confirm that our baseline empirical conclusions regarding core node identification and directional risk transmission dynamics exhibit strong structural robustness and are invariant to threshold calibrations.

## 9. Conclusions and Policy Implications

### 9.1. Empirical Conclusions

By constructing an incremental analysis framework integrating “linear spillovers, nonlinear transmission, and tail risk diffusion”, this study systematically investigates the cross-market risk transmission mechanisms in China from 2004 to 2024. The empirical findings reveal that, distinct from the traditional risk transmission paradigm centered primarily on the banking architecture, the Chinese cross-market risk network exhibits prominent structural characteristics defined as a “technology-driven and real estate-mediated” framework. The specific conclusions are outlined as follows:

First, cross-market risk connectedness displays pronounced pro-cyclicality and a “bad news contagion” effect. The empirical results demonstrate that the Total Connectedness Index (TCI) under downward market regimes is significantly higher than that under upward regimes, exhibiting a distinct “U-shaped” pattern across the conditional quantile distribution. This evidence implies that while individual sub-markets possess a certain degree of risk diversification space under normal market conditions, extreme stress scenarios induce a systemic, nonlinear “all-market resonance”. Consequently, the risk diversification function of traditional asset allocation strategies weakens substantially during extreme volatility, presenting structural failures.

Second, the high-tech market emerges as a consistent net transmitter of nonlinear predictive information within the cross-market network. The analysis grounded in Effective Transfer Entropy (ETE) confirms that the high-tech sector maintains a significant net information-flow attribute. This pattern is consistent with the hypothesis that fluctuations in technology cycles may shape capital market expectations and be propagated to other sectors through nonlinear predictive trajectories. Because ETE captures predictive dependence rather than structural causality, we interpret this finding as suggestive of, rather than conclusive evidence for, a systematic risk spillover mechanism potentially latent within technological innovation cycles, which may complement the traditional cognitive framework that focuses predominantly on financial intermediaries as the primary sources of risk transmission.

Third, the real estate market occupies a crucial “intermediary nexus” position within the cross-market network topology. Complex network metrics reveal that the PageRank centrality of the real estate market consistently ranks at the core level across the sample period. It not only absorbs external risk transfers originating from the energy and high-tech sectors but also functions as a primary intermediary node governing the global risk propagation efficiency of the system. The empirical outputs validate that when the real estate sector undergoes deep structural adjustments, its node vulnerability is highly susceptible to triggering chain reactions, thereby acting as a critical channel and an amplifier that transforms localized sector-specific shocks into systemic contagion.

Fourth, macro exogenous shocks and industrial technological waves constitute the primary exogenous forces driving the structural evolution of the risk network. The dynamic rolling-window analysis indicates that network density registers prominent impulse-type surges during the 2015 domestic equity market turbulence, the 2020 public health emergency, and the 2024 valuation restructurings driven by the Artificial Intelligence (AI) industrial wave. This pattern demonstrates that cross-market connectedness is highly time-varying and co-driven by the macro-institutional environment and industrial cycle transformations, causing the financial system to exhibit distinct structural vulnerability and episodic instability within specific observation windows.

### 9.2. Policy Implications

Based on the aforementioned empirical conclusions, this study proposes the following policy recommendations to effectively mitigate systemic financial risks induced by cross-market linkages:

First, the monitoring of cross-market risk linkages should be significantly strengthened. In light of the heterogeneous roles played by the high-tech and real estate markets within the network topology, regulatory authorities should dismantle sectoral “information silos”. Utilizing complex network metrics—such as PageRank centrality and directional ETE flows—a dynamic risk early-warning architecture and a cross-market linkage monitoring framework should be established. Regulatory focus should be directed toward monitoring the feedback effects of valuation fluctuations in the high-tech sector on the broader financial system, as well as tracking the stress-bearing thresholds of the real estate market as a critical node, thereby achieving early identification and dynamic tracking of cross-market risks.

Second, differentiated and dynamic macroprudential supervision strategies should be implemented. For the high-tech sector, which exhibits leading driver characteristics, regulators should guide rational capital allocation to prevent excessive financialization of technological concepts from inducing localized asset bubbles. For the real estate market, which occupies a central intermediary position, it is imperative to enhance its node resilience under extreme stress conditions by optimizing risk provisioning and liquidity buffer mechanisms, thereby preventing it from transforming from a systemic risk absorber into a net risk transmitter.

Third, cross-market emergency resolution and hedging mechanisms under extreme stress scenarios should be optimized. Given that risk linkages exhibit explosive nonlinear acceleration under extreme quantile conditions, regulatory bodies should institutionalize collaborative cross-market stress testing frameworks and refine cross-departmental risk-sharing and coordinated resolution mechanisms. Simultaneously, the depth and breadth of the domestic debt market should be further enhanced to strengthen its risk-hedging functionality within the financial architecture, guiding orderly capital flows during crisis periods and effectively reducing the global coupling intensity of the systemic network.

Fourth, expectation management regarding financial volatility associated with “technology cycles” should be enhanced. Regulators must closely monitor asset revaluation risks that may accompany emerging technological waves while driving real economic growth. By improving market transparency and strengthening information disclosure requirements, authorities should guide investors to evaluate technological innovations from a long-term value perspective. This approach will help suppress irrational cross-market contagions triggered by sentiment resonances, safeguarding the stable operation of the financial system during periods of economic momentum transition.

### 9.3. Limitations and Future Research

This study is subject to several limitations that warrant acknowledgment. First, constrained by data availability, the empirical analysis relies on aggregate market-level indices, which do not incorporate the heterogeneous risk characteristics latent within micro-level financial institutions and firms. Future research could incorporate micro-structural data to enhance the granular explanatory power of the model. Second, the symbolic discretization procedure inherent in the ETE methodology may affect the measurement precision of nonlinear information flows. Future extensions could combine high-frequency financial data, TVP-VAR specifications, or multilayer network frameworks to more accurately capture the dynamic evolutionary mechanisms and microfoundations of cross-market risk propagation. Third, the ETE estimates reported in this paper measure directional predictive dependence and do not identify structural causal effects. Future research could exploit exogenous shocks—such as identifiable technology-policy announcements or geopolitical events—and employ event-study or instrumental-variable designs to strengthen the causal interpretation of the nonlinear information flows documented here.

## Figures and Tables

**Table 1:** Proxy variables and data sources for the six core markets.

Market	Proxy Variable	Data Source
Stock	CSI 300 Index	Wind, CSMAR
Debt	China Bond Composite Wealth Index	Wind, China Bond Information Center
Forex	USD/CNY Exchange Rate	China Foreign Exchange Trade System
Real Estate	Shenwan Real Estate Index	Wind
Energy	CSI All Share Energy Index	Wind
High-tech	Paasche composite of five high-tech sub-sector indices	Calculated from Wind/CSI indices

**Table 2:** Descriptive statistics of returns across the six core markets.

Market	Mean	SD	Min	Max	Skewness	Kurtosis	JB *p*-Value
Stock	0.0209	1.3995	−5.0600	3.8808	−0.4855	5.1303	0.0000
Debt	0.0173	0.0658	−0.1969	0.2359	0.0204	5.3036	0.0000
Forex	−0.0019	0.1737	−0.6312	0.5805	−0.0531	6.2960	0.0000
Real Estate	0.0093	2.0031	−7.0521	5.3929	−0.4053	4.9138	0.0000
Energy	0.0217	1.8446	−5.7736	5.1799	−0.2140	4.2948	0.0000
High-tech	0.0444	2.9433	−7.6003	7.6861	0.0028	3.1687	0.0559

**Table 3:** ADF unit root test results.

Market	ADF Statistic	*p*-Value	Stationarity
Stock	−15.0986	0.01	Yes
Debt	−13.1260	0.01	Yes
Forex	−14.3021	0.01	Yes
Real Estate	−16.4929	0.01	Yes
Energy	−15.9682	0.01	Yes
High-tech	−17.5180	0.01	Yes

**Table 4:** ARCH-LM effect test results.

Market	ARCH Statistic	*p*-Value	ARCH Effect
Stock	727.3651	0.0000	Yes
Debt	857.0137	0.0000	Yes
Forex	862.1203	0.0000	Yes
Real Estate	830.9718	0.0000	Yes
Energy	545.2788	0.0000	Yes
High-tech	263.2441	0.0000	Yes

**Table 5:** Correlation matrix of returns across the six core markets.

Market	Stock	Debt	Forex	Real Estate	Energy	High-Tech
Stock	1.0000	−0.0544	−0.1410	0.7800	0.8325	0.5050
Debt	−0.0544	1.0000	0.0524	−0.0517	−0.0535	−0.0492
Forex	−0.1410	0.0524	1.0000	−0.0968	−0.1000	−0.0821
Real Estate	0.7800	−0.0517	−0.0968	1.0000	0.6372	0.4398
Energy	0.8325	−0.0535	−0.1000	0.6372	1.0000	0.3777
High-tech	0.5050	−0.0492	−0.0821	0.4398	0.3777	1.0000

**Table 6:** Static risk spillover matrix across the six core markets (%).

Receive\Transmit	Stock	Debt	Forex	Real Estate	Energy	High-Tech	FROM
Stock	38.63	0.16	0.80	23.61	26.71	10.09	61.37
Debt	0.93	96.61	0.56	0.70	0.96	0.24	3.40
Forex	1.99	0.36	94.77	1.03	1.08	0.77	5.23
Real Estate	27.40	0.19	0.48	44.65	18.15	9.12	55.35
Energy	30.71	0.15	0.48	18.05	44.15	6.46	55.85
High-tech	15.88	0.17	0.52	12.31	8.92	62.20	37.81
TO	76.92	1.03	2.84	55.72	55.82	26.68	TCI
NET	15.54	−2.36	−2.39	0.37	−0.03	−11.13	36.50%

**Table 7:** Dynamic net spillover statistics for the six core markets (%).

Market	Dynamic NET Mean	Static NET	Role Orientation
Stock	16.10	15.54	Core Transmitter
High-tech	−8.59	−11.13	Receiver → Transmitter
Debt	−5.75	−2.36	Stable Receiver
Forex	−5.48	−2.39	Receiver
Energy	3.12	−0.03	Receiver → Transmitter
Real Estate	0.57	0.37	Neutral

**Table 8:** Summary of static risk spillover asymmetry analysis.

Market	Positive Net Spillover (*NET*^+^)	Negative Net Spillover (*NET*^−^)	Asymmetric Impact (*Diff*)
Stock	14.42	13.00	−1.42
Real Estate	−2.41	1.14	3.55
Energy	−1.56	−0.28	1.27
Bond	−0.46	−0.65	−0.19
Forex	−1.12	−1.17	−0.06
High-tech	−8.87	−12.03	−3.16

**Table 9:** Comparison of network topological metrics under different shock structures.

Topological Metric	Positive Network (*r*^+^)	Negative Network (*r*^−^)	Percentage Change
Network Density	0.4000	0.4000	0.00%
Active Edges	12.00	12.00	0.00%
Average Weight	14.49	17.46	+20.44%
Average Path Length	0.0924	0.0722	−21.86%
Clustering Coefficient	1.0000	1.0000	0.00%

**Table 10:** Cross-market Effective Transfer Entropy (ETE) matrix.

Market Class	Stock	Debt	Forex	Real Estate	Energy	High-Tech
Stock	0.0000	0.0017	0.0016	0.0059	0.0015	0.0031
Debt	0.0022	0.0000	0.0031	0.0010	0.0021	0.0023
Forex	0.0024	0.0012	0.0000	0.0031	0.0021	0.0025
Real Estate	0.0042	0.0023	0.0028	0.0000	0.0025	0.0055
Energy	0.0014	0.0019	0.0019	0.0026	0.0000	0.0018
High-tech	0.0031	0.0019	0.0021	0.0052	0.0018	0.0000

**Table 11:** Market centrality metrics and role orientations within the ETE backbone network.

Market Segment	Out-Degree	In-Degree	Net Flow	PageRank	Risk Role Orientation
High-tech	0.0156	0.0037	0.0118	0.0529	Net Risk Transmitter
Energy	0.0069	0.0033	0.0036	0.0483	Net Risk Transmitter
Real Estate	0.0042	0.0163	−0.0121	0.4319	Net Risk Receiver
Stock	0.0059	0.0092	−0.0033	0.4197	Net Risk Receiver
Debt	0.0031	0.0032	−0.0001	0.0236	Neutral Role
Forex	0.0032	0.0033	−0.0001	0.0236	Neutral Role

**Table 12:** Robustness check: Comparison of network metrics across alternative percentile thresholds.

Threshold	ETE Critical Value	Edges	Network Density	Core Nexus (Top PR)	Primary Risk Source
70%	0.00313	9	30.00%	Real Estate	High-tech
75%	0.00318	8	26.67%	Real Estate	High-tech
80%	0.00350	6	20.00%	Real Estate	High-tech

Note: The core nexus is identified based on PageRank centrality, and the primary risk source is evaluated via the Net Flow metric.

## Data Availability

Data were obtained from Wind (https://www.wind.com.cn/, accessed on 20 May 2026), CSMAR (https://data.csmar.com/, accessed on 25 May 2026), China Bond Information Center (https://www.chinabond.com.cn/, accessed on 28 May 2026), China Foreign Exchange Trade System (https://www.chinamoney.com.cn/, accessed on 1 June 2026), and are available from these sources with the permission of the respective third parties. The high-tech Paasche index was calculated from five Wind and CSI sub-sector indices (semiconductor, aerospace and military, communications, computer, and pharmaceutical manufacturing).
